# The global outbreak of Oropouche virus: surveillance of global trends

**DOI:** 10.1186/s40794-026-00297-0

**Published:** 2026-03-28

**Authors:** Rahul Sharma, Khyati Sharma, Balamurugan Balusamy, Rishabha Malviya, Aarthi Sivasankaran, Sonali Sundram

**Affiliations:** 1 Department of Pharmacy, School of Medical and Allied Sciences, Galgotias University, Greater Noida, Uttar Pradesh India; 2 School of Engineering and IT, Manipal Academy of Higher Education, Dubai Campus, Dubai, UAE; 3 Google Inc., Seattle, WA USA

**Keywords:** Oropouche virus, Arbovirus, Vector-borne disease, Emerging infectious disease, *Culicoides paraensis*, Surveillance, Diagnostics

## Abstract

Oropouche virus (OROV) is a re-emerging arthropod-borne virus of the genus *Orthobunyavirus* (family *Peribunyaviridae*) that poses an increasing public health concern in South and Central America and the Caribbean. First identified in Trinidad in 1955, OROV has caused recurrent outbreaks, with recent resurgence associated with geographic expansion and increased urban transmission. In 2024 alone, more than 11,600 confirmed cases were reported in the Region of the Americas, with continued transmission documented in early 2025. The virus is primarily transmitted in urban settings by the biting midge *Culicoides paraensis*, while other mosquito species have demonstrated variable laboratory vector competence. Clinically, OROV infection presents as an acute febrile illness resembling dengue, chikungunya, and other arboviral infections, frequently complicating diagnosis in co-endemic areas. Although molecular diagnostics such as RT-PCR enable early detection, standardized diagnostic protocols and validated commercial assays remain limited. No specific antiviral therapy or licensed vaccine is currently available, and prevention relies on vector control and personal protective measures. Emerging evidence has raised concern regarding potential vertical transmission of OROV during pregnancy. Case reports and recent genomic analyses have described associations between maternal infection and adverse fetal outcomes, including stillbirth, congenital microcephaly, and structural anomalies. Detection of viral RNA in placental or fetal tissues supports biological plausibility; however, systematic cohort-based epidemiological data remain limited. At present, causality cannot be definitively established, and further prospective surveillance studies are urgently needed to clarify risk magnitude and pathogenic mechanisms. This review synthesizes current knowledge on the epidemiology, virology, transmission dynamics, clinical manifestations, diagnostics, and prevention strategies of OROV and identifies key research priorities to strengthen surveillance, preparedness, and outbreak response in affected and at-risk regions.

**Graphical Abstract:**

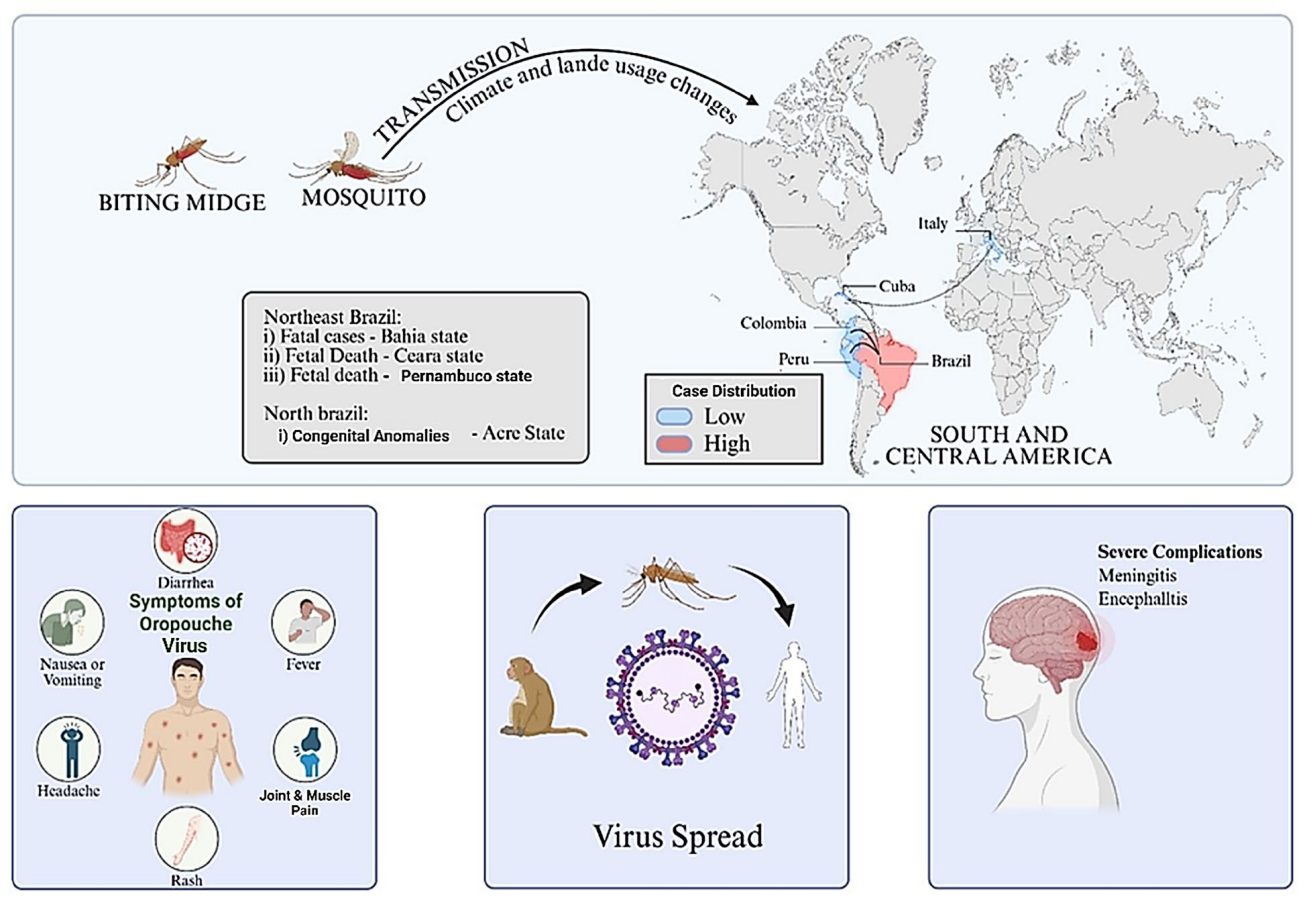

## Introduction

The Oropouche virus (OROV), a segmented single-stranded RNA virus that is a member of the genus *Orthobunya* virus within the *Peribunyaviridae* family, is the arboviral agent that causes the arboviral agent that causes Oropouche fever. South America, Central America, and the Caribbean have all reported cases of the virus. Since it was first discovered by a forestry worker in Trinidad in 1955, there have been other notable human outbreaks and epidemics reported in the Americas [1, 2]. There have been 11,634 confirmed cases of Oropouche, including two fatalities, in the ten countries and one territory in the Region of the Americas: Colombia, Cuba, Ecuador, Guyana, Panama, Peru, Brazil, Canada, the Cayman Islands, and the United States. OROV is primarily transmitted to humans through the bite of infected bitten by a *Culex quinquefasciatus* mosquito or the *Culicoides paraensis* midge, which is found in wooded regions and near bodies of water. *Culicoides paraensis* is the primary confirmed urban vector; Culex quinquefasciatus has demonstrated laboratory competence of the urban cycle. Other mosquito species that may contribute to the OROV transmission cycle include *Coquillettidia venezuelensis*,* Aedes serratus*, and *Culicoides paraensis.* [2, 3]. OROV’s tri-segmented genome is prone to reassortment, which could lead to new viruses when co-infected with genetically similar viruses. Previously, three OROV reassortants were identified: Perdões, Madre de Dios, and Iquitos. Human febrile illnesses have been associated with Madre de Dios and Iquitos viruses [4]. Symptoms resemble dengue and typically begin four to eight days (range 3–12 days) after the infective bite (between three to 12 days) after the infective bite. The onset is sudden, usually with fever, intense headache, joint stiffness, pain, chills, and sometimes persistent nausea and vomiting, for up to seven days [5]. Up to 60% of cases have a relapse of symptoms after the fever stops. Most cases recover within seven days, however, in some patients, convalescence can take weeks. Severe clinical presentation is rare, but it may result in aseptic meningitis during the second week of the disease [2, 6]. For diagnostic purposes, Real-time PCR is the primary diagnostic method for detecting OROV infection. For clinical diagnosis, saliva and urine samples may identify viral RNA more accurately than serum samples. Additional testing includes assays for viral isolation and plaque reduction neutralisation [7, 8]. Oropouche virus sickness has no known cure or vaccination, and sufferers are only able to recover with supportive care. The main strategies for slowing the virus’s transmission are vector control and personal protective equipment. Personal protective measures, such as donning protective clothes and applying insect repellents that contain DEET, IR3535, or icaridin, are advised as preventive measures to reduce the chance of infection [6]. Though animal models for possible vaccinations are scarce, ribavirin, mycophenolic acid, and IFN-α have been tested in mice models with varying results against OROV.

## Epidemiology

For several decades, the Oropouche virus (OROV), which was initially identified in Trinidad and Tobago in 1955, produced intermittent outbreaks, mostly in the Amazonian regions of Brazil and its adjacent nations. Its geographic distribution was restricted, and the number of cases was relatively modest. Periodically, significant outbreaks have been reported in Brazil, Panama, Peru, and other regions of South and Central America; historical totals are thought to have reached hundreds of thousands of infections during the course of the late 20th and early 21st centuries [9–11].

A marked epidemiological shift in Oropouche virus (OROV) transmission was observed toward the end of 2023, characterized by geographic expansion and a substantial increase in reported cases. This rise likely reflects a combination of intensified clinical surveillance and improved diagnostic capacity, along with the introduction and sustained transmission of OROV in previously unaffected regions. In 2024, more than 11,600 laboratory-confirmed cases were reported across the Region of the Americas, including Brazil, Bolivia, Peru, Colombia, Cuba, Ecuador, Guyana, and Panama. This represents a significant increase compared with pre-2023 historical baseline levels, highlighting the expanding public health importance of OROV in the Americas Recent evidence indicates that Oropouche virus (OROV) infection is no longer restricted to the Amazon Basin but has expanded into peri-urban and urban regions, with increasing reports from previously non-endemic areas. Comparative analyses of past outbreaks demonstrate a clear epidemiological shift characterized by geographic expansion and a rise in laboratory-confirmed cases. This trend has been attributed to improved molecular diagnostics, strengthened surveillance, environmental changes, urbanization, and potential shifts in vector distribution. These developments highlight the need for enhanced surveillance and coordinated public health response strategies.

According to the World Health Organization (WHO) Disease Outbreak News reports and Pan American Health Organization (PAHO) Epidemiological Updates, as of 30 April 2025, a cumulative total of 11,634 laboratory-confirmed cases of Oropouche virus disease have been reported in the Region of the Americas since the beginning of the recent expansion in late 2023 [12–15].*“Confirmed cases”* refer to infections verified by molecular detection (RT-PCR or RT-qPCR) or validated serological criteria as defined by national surveillance systems and reported to PAHO/WHO. *“Reported cases”* may include suspected and probable cases depending on national reporting frameworks, whereas *“imported cases”* refer specifically to laboratory-confirmed infections acquired outside the reporting country and identified through travel history investigation.

**Fig. 1:**
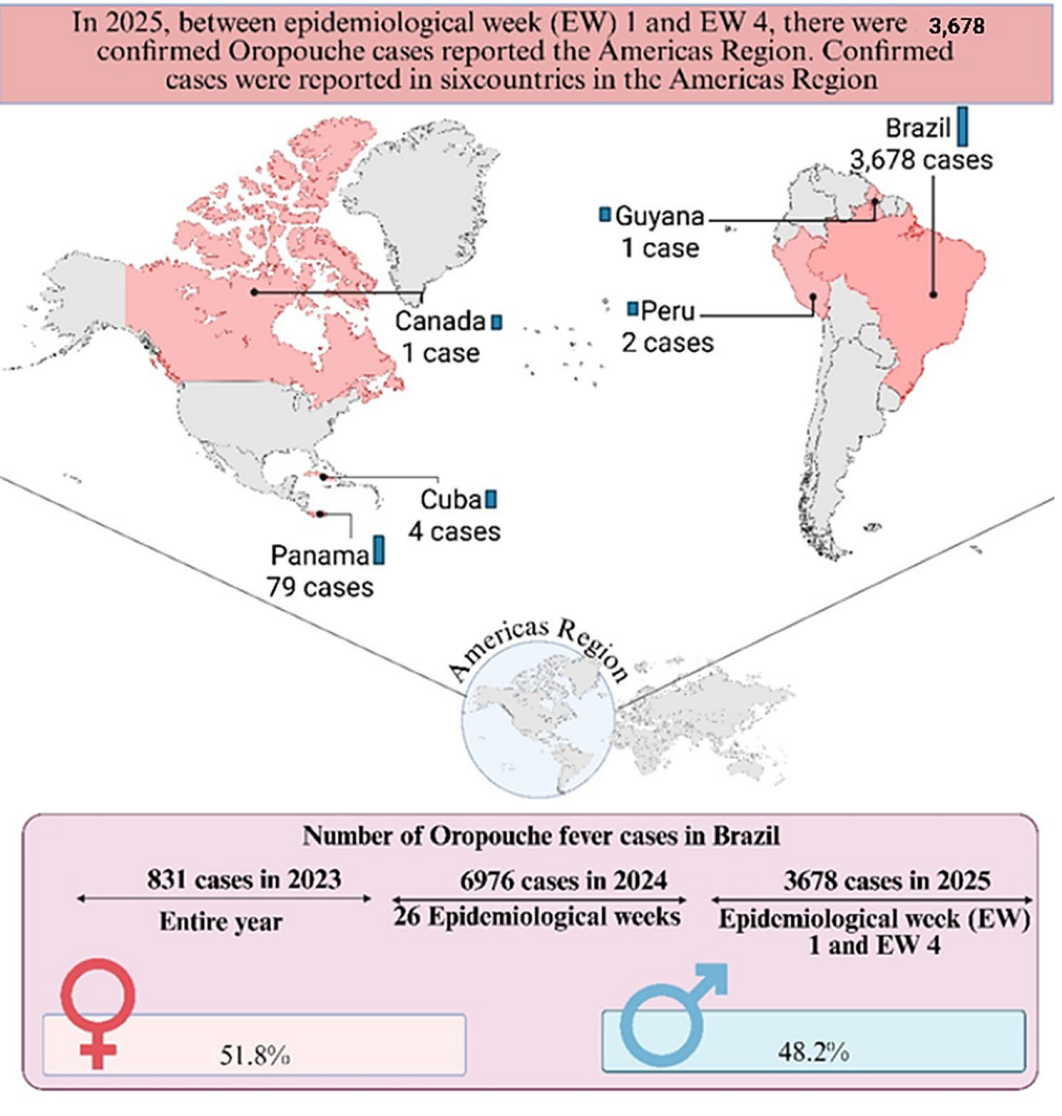
Oropouche fever cases in the Americas (2025). Image Created with Biorender Tool

During 2024 alone, more than 11,600 confirmed cases were reported across multiple countries in the Americas, representing a substantial increase compared to historical baseline incidence prior to 2023. Autochthonous transmission has been documented primarily in Brazil, Peru, Bolivia, Colombia, Cuba, Ecuador, Guyana, and Panama. In addition, travel-associated imported cases have been reported in North America and Europe, including Canada and several European countries, reflecting increasing international spread [16, 17]. Between January and April 2025, 3,678 laboratory-confirmed cases were reported in Brazil, representing the largest national burden during that reporting period. These figures should be interpreted in the context of evolving surveillance capacity, increased diagnostic awareness, and expansion of molecular testing infrastructure in affected regions [17]. The illustration of the case is provided in Fig. 1.

Importantly, case counts may underestimate the true incidence of infection due to clinical overlap with other arboviral diseases such as dengue and chikungunya, limited laboratory availability in rural areas, and differences in national case definitions and reporting completeness [12–15, 18].

The map displays confirmed instances of Oropouche (EW 1–4) in six different countries, with Brazil reporting the most occurrences (3,678). Brazil’s case counts in 2023 and 2025 are shown in the chart below, with a small female majority in 2025 (51.8%).

## Virology and transmission

The schematic shows the virion structure (~ 70 nm) with surface glycoproteins (Gn, Gc), ribonucleocapsid (RNP), and RNA segments: L (RdRp), M (glycoproteins), and S (nucleoprotein). Each segment encodes essential viral proteins for replication and host interaction.

### Virus characteristics and genome organization

**Fig. 2:**
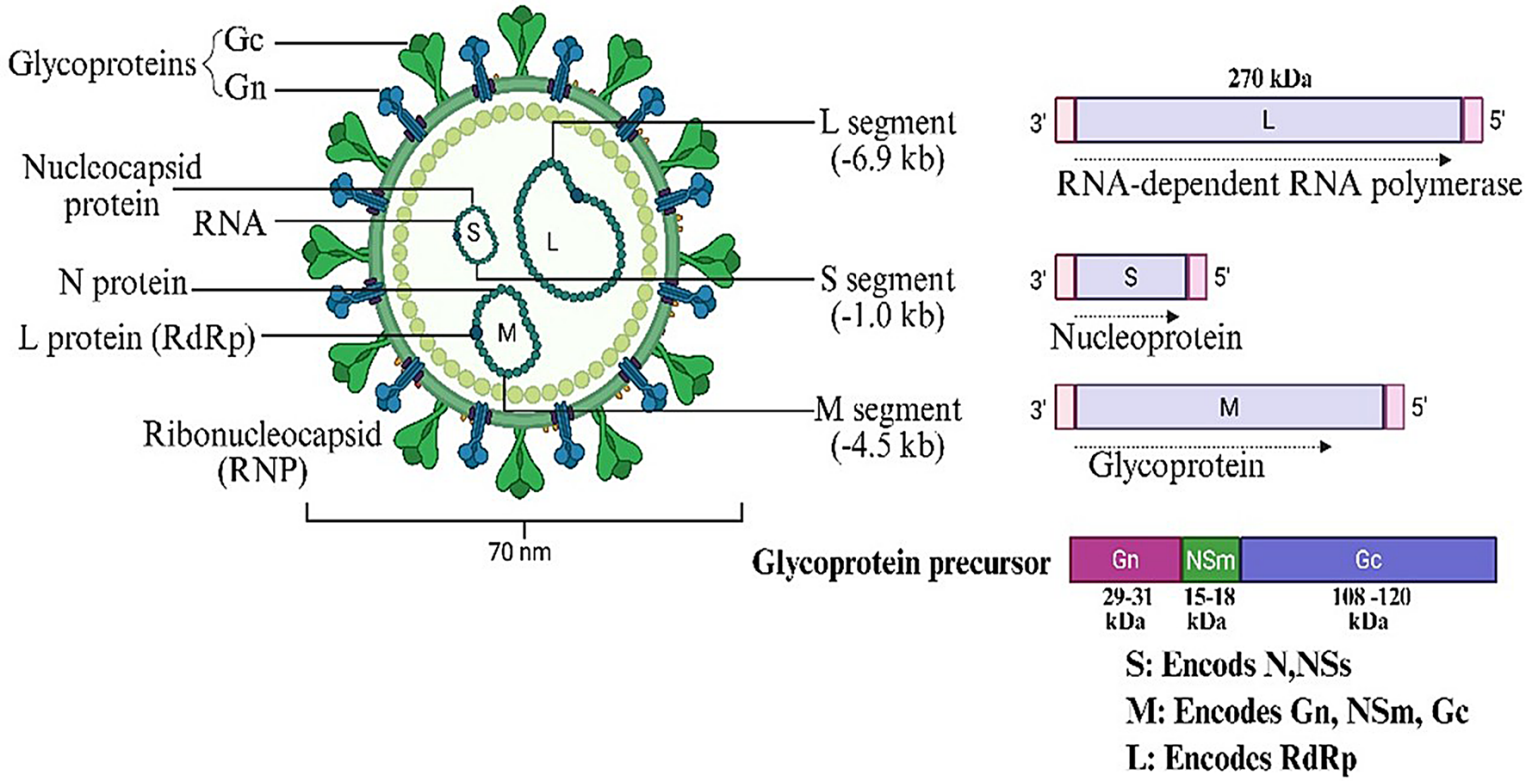
Structure and genome organization of the Oropouche virus (OROV). Image Created with Biorender Tool

OROV, a bunyavirus belonging to the Simbu serogroup within the genus *Orthobunyavirus* [4], order *Bunyavirales*, and family *Peribunyaviridae*, possesses an enveloped structure and a segmented genome composed of three negative-sense, single-stranded RNA segments [19]. The small (S) segment encodes two overlapping open reading frames (ORFs) that produce the nucleocapsid protein (N) and the nonstructural protein NSs, the latter of which acts as an inhibitor of type I interferon. The medium (M) segment encodes a polyprotein that is processed post-translationally into the structural glycoproteins Gn and Gc, as well as the lesser-understood NSm protein [20, 21]. Gc, a protein of 939 amino acids, has three to four potential N-linked glycosylation sites, while Gn, a class II membrane fusion protein with 290 amino acids, contains one predicted N-linked glycosylation site. In Schmallenberg virus (SBV), the N-terminal region of Gc includes an extended multi-domain structure with two β-sheet subdomains linking the α-helical head domain to the stalk. Notably, this is the only high-resolution crystal structure available for Gn and Gc among Orthobunyavirus members. Nevertheless, structural data exist for the α-helical head domains of Gc from BUNV, OROV, and La Crosse virus (LACV), indicating conservation of this α-domain across these viruses. They are believed to have a single N-linked glycosylation site exposed to solvent. Based on available data, it is believed that Gc and Gn combine to produce a trimeric spike complex that protrudes from the viral envelope. However, as no monoclonal antibodies have been developed against OROV, it is unclear where the epitopes important in neutralization and protective immunity are located. However, Gn for SBV has been mapped to the epitopes linked to protective immunity [22, 23]. There are no known OROV cell receptors. Orthobunya viruses’ segmented genomes are a key genetic characteristic that allows them to quickly reassort when two viruses with similar genetic makeup infect the same cell. In the evolution of bunyaviruses, this reassortment ability is a potent process that allows for the spontaneous creation of new viral strains and species with unique characteristics [24]. The L protein and an RNA-dependent RNA polymerase are encoded by the large (L) segment [25]. The untranslated regions (UTRs) that encircle the coding sequences of each segment are crucial for transcription, packaging, and replication. According to research on other Orthobunya viruses, the enclosed OROV virion is around 90 nm in diameter, contains three ribonucleoprotein (RNP) complexes, and exhibits Gc and Gn on its surface. As seen in (Fig. 2), each of these complexes is composed of RNA segments complexed with several copies of the N and L proteins. In contrast, there are not many high-resolution structural studies on OROV [26]. The Orthobunya virus category includes the Oropouche virus. RNA is a type of genetic material found in the Oropouche virus. The portion, medium part, and large part are the three components that make up this RNA.

The Oropouche virus can exchange portions of its material with other Oropouche viruses infecting the same cell. Reassortment has been shown to play a part in the establishment and geographic spread of divergent strains in naturally occurring OROV lineages and related Simbu serogroup viruses. Because antigenic alterations may lessen cross-protective immunity and make it more difficult to formulate broadly effective immunogens, such genetic plasticity poses difficulties for vaccine development and immunological recognition [9].

### Role of vectors and reservoir hosts

**Fig. 3:**
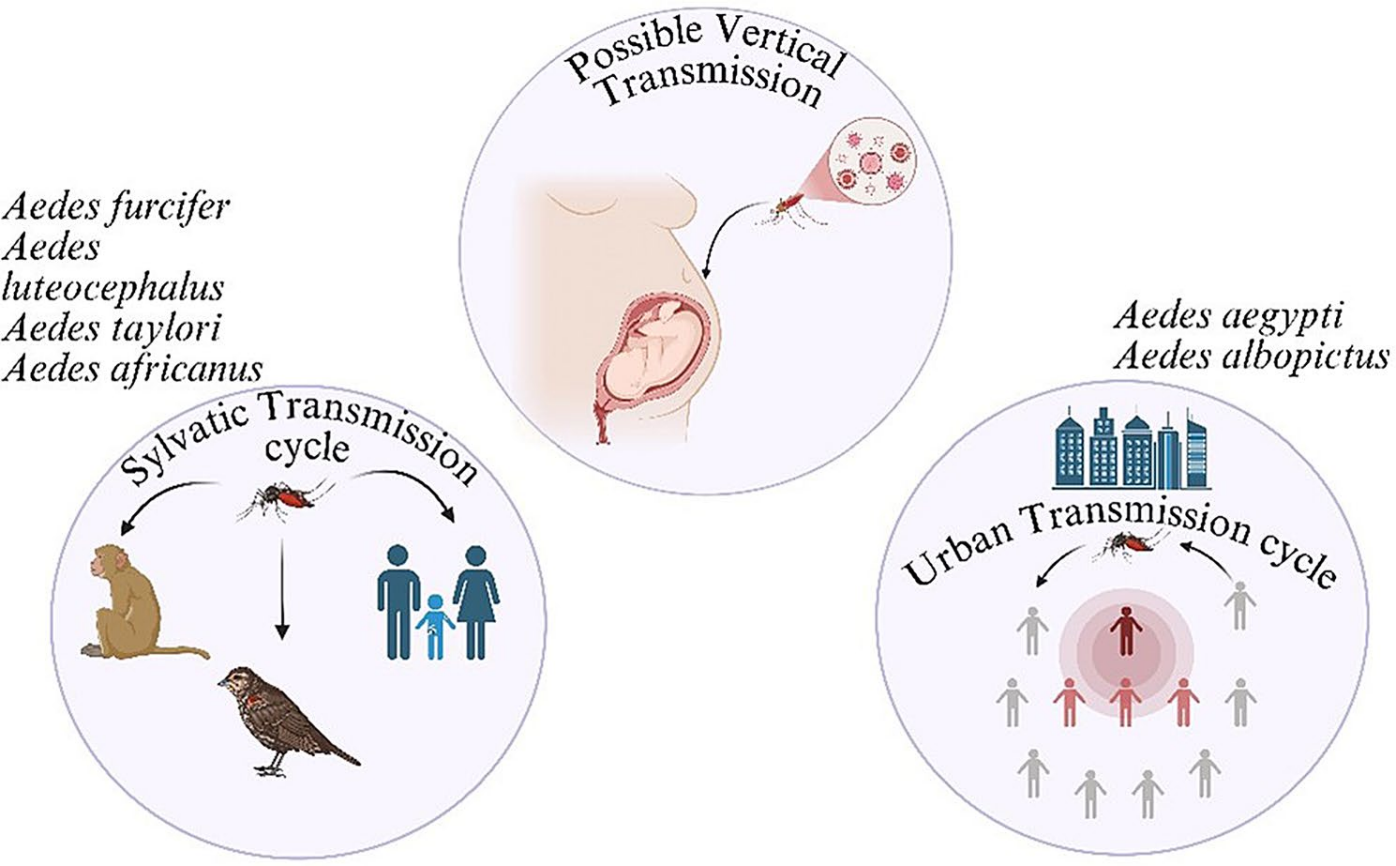
Possible, Sylvatic and urban transmission cycles of OROV. Image Created with Biorender Tool

*Culicoides paraensis* remains the primary confirmed vector responsible for urban transmission of OROV, supported by repeated field isolation and epidemiological correlation during outbreaks in Brazil and neighboring countries. In contrast, the sylvatic transmission cycle remains less clearly defined. While vertebrate hosts such as nonhuman primates and sloths have been implicated, the definitive arthropod vector responsible for maintaining sylvatic transmission has not been conclusively identified. The virus is spread by many vectors and animal reservoirs in both sylvatic (forest) and urban cycles, which are represented in Fig. 3. In addition to the primary confirmed vector *Culicoides paraensis*, several mosquito species depicted in Fig. 3-including *Culex quinquefasciatus*,* Aedes serratus*,* Aedes aegypti*,* Aedes furcifer*, and *Aedes taylori*-have been implicated as suspected or experimentally evaluated vectors of Oropouche virus [27]. Evidence for these species is largely derived from laboratory vector competence studies or limited field detections, and their role in sustained human transmission remains unconfirmed. These species are therefore presented to illustrate potential transmission pathways rather than established epidemiological drivers of OROV outbreaks [28, 29]. *Culex quinquefasciatus*,* Coquillettidia venezuelensis* and *Aedes serratus* mosquitoes can also act as possible vectors [3]. It is thought that the virus spreads through both an urban epidemic cycle between insects and humans and a sylvatic cycle in wooded areas. Vertebrate hosts in the sylvatic cycle include nonhuman monkeys, sloths, and maybe even birds [6] In the sylvatic transmission cycle, the definitive arthropod vector has not been conclusively identified; however, in the urban transmission cycle, *Culicoides paraensis* has been repeatedly implicated based on field isolation and epidemiologic evidence While *Culicoides paraensis* is well-supported as the primary urban vector in endemic regions, the vector(s) responsible for transmission in newly affected geographic areas remain under investigation. Considering that the virus is spreading to formerly unexplored areas (such as Cuba) and could potentially reach new territories (like the US), it is imperative to comprehend the potential transmission routes and threats posed by the broad variety of vectors Prior to 2024, relatively few experimental vector competence studies had systematically evaluated transmission efficiency across geographically diverse vector populations. Recent investigations have begun addressing this gap, highlighting the importance of expanded entomological surveillance in newly affected regions [30].

The OROV virus is primarily transmitted to people in urban areas by insects, specifically *Culicoides paraensis* midges. The virus can also be carried by other midges, such as C. sonorensis.

*Culex quinquefasciatus*,* Aedes serratus*, and *Coquillettidia venezuelensis* mosquitoes are less effective at dispersing the virus. Both in lab experiments and in real life, researchers have observed that they are not particularly good at spreading the virus. For epidemic preparedness and focused vector management methods, it is crucial to distinguish between suspected and proven vectors, such as *Culicoides paraensis* [27].

## Clinical features and diagnosis

### Clinical symptoms

Oropouche virus infection clinically resembles other arboviral infections, such as the dengue virus and the chikungunya virus. There are some things that can help doctors tell the Oropouche virus infection apart from these other diseases particularly in outbreak settings with limited diagnostic resources are available. Oropouche virus infections typically begin abruptly with chills, a very high fever, severe headache and joint and muscle pain [31]. Individuals infected with the Oropouche virus frequently experience stomach issues, including nausea, vomiting, stomach discomfort, and difficulty swallowing. The Oropouche virus infection is a sickness that can make patients feel quite sick. Compared to dengue, OROV infection is less likely to cause bleeding issues or low platelet counts [32]. When an OROV infection appears to be improving, symptoms frequently return. Approximately 60–70% of patients experience relapse of symptoms [31].

OROV infection is different from chikungunya in that people with OROV do not have long lasting joint pain that lasts for months. When doctors do tests on people, with OROV they often find that their white blood cell counts go down for a while or they have slightly elevated liver enzymes. If someone has low platelet counts it is more likely they have dengue [31]. If their platelet counts are normal and their joints are very inflamed it is more likely they have chikungunya. Although these features are not diagnostic in isolation, their combined assessment may help raise early suspicion for OROV in co-endemic regions and guide appropriate molecular or serological testing for confirmation [33] and comparative table is been discussed in (Table 1).

Oropouche sickness incidents often occur predictably, with the rainy season accounting for the majority of cases. The pathogenic virus first proliferates in nearby cells, including macrophages, endothelial cells, and epithelial cells, resulting from a bite from a mosquito or biting midge. Following that, it enters the infected person’s bloodstream, resulting in viremia and OROV symptoms. The clinical presentation of OROV often resembles that of other arboviral diseases, notably dengue, chikungunya, and Zika. Following a 3–10 day incubation period, patients usually have fever, chills, headache, myalgia, and arthralgia all at once. Abdominal pain, maculopapular rash, photophobia, vomiting, diarrhoea, exhaustion, retroorbital pain, and conjunctival injection are possible additional symptoms. Although the initial symptoms typically resolve within a few days, as many as 70% of individuals experience symptoms that resurface for several weeks after the initial illness has subsided [34, 35].

The pathogen was found in significant quantities in the brain, liver, and blood of golden hamsters infected with OROV, but it was absent in other organs. In histopathological reports, hepatitis and meningoencephalitis were found, and the liver and brain had high levels of OROV antigen [36]. OROV fever patients had slightly elevated liver enzymes, although there is no solid evidence that it causes liver diseases. Infected persons may have rash, anorexia, retro-orbital pain, and hemorrhagic indications. With viral titers ranging from 103 to 107 infectious doses per millilitre of serum, many patients evaluated in the initial days of clinical illness are viremic. By feeding on blood with these viral levels, naïve female midges can become infected, supporting the hypothesis that humans are the exclusive amplifying host in urban transmission cycles. In some neuroinvasive situations, OROV has been detected not only in blood but also in the cerebrospinal fluid (CSF) [37]. CNS infections leading to meningoencephalitis or aseptic meningitis, though seemingly uncommon, represent serious clinical manifestations of the virus [28].

**Table 1:** Clinical and Laboratory Features Useful for Differentiating Oropouche Virus Infection from Dengue and Chikungunya

Feature	Oropouche Virus (OROV)	Dengue Virus (DENV)	Chikungunya Virus (CHIKV)	References
Fever onset	Sudden, high-grade	Sudden, high-grade	Sudden, high-grade	[29, 38]
Headache	Common, often severe	Very common	Common	[29, 39]
Arthralgia	Common, usually transient	Mild–moderate	Severe, often persistent	[38]
Myalgia	Common	Prominent	Common	[29, 40]
Rash	Variable, less frequent	Common	Common	[29, 38]
Gastrointestinal symptoms	Frequent (nausea, vomiting, abdominal pain)	Common	Less prominent	[39]
Hemorrhagic manifestations	Rare	Relatively common in severe disease	Rare	[29, 40]
Relapse of symptoms	Common (up to 60–70%)	Uncommon	Uncommon	[39]
Leukopenia	Common	Common	Variable	[29, 40]
Thrombocytopenia	Mild or absent	Often marked	Usually mild or absent	[29, 38]
Liver enzyme elevation	Mild	Moderate–severe	Mild	[38, 40]
Chronic joint symptoms	Rare	Rare	Common	[38, 40]
Key diagnostic clue	Symptom relapse, GI features, mild cytopenias	Severe thrombocytopenia, bleeding	Persistent disabling arthralgia	[29, 38]

### Advances in diagnostic approaches

It is quite difficult to diagnose the Oropouche virus infection. This is due to the fact that the symptoms of the Oropouche virus are not highly specific and may resemble those other viruses that are prevalent in the same regions, such as dengue and chikungunya. We must perform tests, such as molecular and serological testing, to determine whether a person has the Oropouche virus. The test we select and its efficacy for the Oropouche virus will depend on when we gather the samples and how effectively the local laboratory performs.

Molecular Methods: Researchers used something called reverse transcription polymerase chain reaction or RT-PCR for also quantitative RT-PCR, which is RT-qPCR to detect OROV RNA early on. These methods are like the gold standard. They are very good at finding OROV RNA in blood samples taken in the week that someone is sick. The assay’s 95% LLOD was 5.6 copies/µL (H75 strain) and 10.8 copies/µL (BeH strain). All samples > 10 copies/µL were detected; 8/14 at ≤ 10 copies/µL. A “tiny drop” (~ 5 µL blood) thus contains ~ 25–50 copies total, enabling detection even at low viremia [41]. RT-PCR and RT-qPCR assays remain the reference molecular methods for detection during the acute viremic phase. Reported limits of detection vary between assay designs, and direct comparison across studies remains difficult due to lack of standardized validation criteria [17]. This aids in differentiating OROV from related viruses. RT-qPCR is particularly useful for detecting OROV when it is not prevalent because it searches for portions of the virus that do not change substantially. RT-PCR utilization is frequently restricted to central laboratories in resource-constrained settings due to the absence of specialized equipment, qualified workers, and decentralized testing facilities, which can delay results and hinder accessibility. For field or point-of-care testing, alternative molecular assays such reverse-transcription loop-mediated isothermal amplification (RT-LAMP) have shown similar sensitivity to RT-PCR while requiring no infrastructure and offering quick visual findings.

Serological Methods: By identifying host antibody responses, serology can help with diagnosis, especially following the acute viremic period. IgG responses increase after IgM antibodies are usually evident 4–7 days after initiation. Although they need paired samples and specialist laboratories, plaque reduction neutralization tests (PRNT) continue to be the serological “gold standard” for confirming infection and differentiating OROV from comparable arboviral antibodies. The lack of widely validated commercial kits and possible cross-reactivity with antibodies to other circulating arboviruses limit the utility of enzyme-linked immunosorbent assays (ELISA) for detecting IgM/IgG in research settings [29, 42].

RT-PCR and RT-qPCR testing should be used during the week when individuals are ill with a rapidly spreading disease and researchers are short on resources. Following that, researchers are unable to perform those tests; instead, they can employ PRNT tests and assays like IgM and IgG to screen for antibodies [43, 44].

Practical Considerations: RT-PCR and RT-qPCR are important considerations for researchers. Take into account the other tests, such as PRNT, IgM, and IgG, as well as when they will be most helpful. In endemic areas, investments in decentralized molecular testing platforms and the validation of quick, field-adapted assays like RT-LAMP can significantly improve surveillance, response, and epidemic detection [42].

During epidemics in remote or rural areas, quick OROV detection is crucial. Therefore, a transportable SmartCyclerTM [45] was used to establish a one-step RT-qPCR approach for testing for orthobunyaviruses. If RT-PCR methods fail to identify divergent OROV strains due to their high target sequence specificity, they should be used with viral antigen detection or serological testing as part of an orthogonal testing approach. In circumstances where alternative RT-qPCR technologies might not recognise viral genome RNA, metagenomic analysis of samples from patients has been employed to discover and characterise distinct reassorted or unknown viral strains [46], which can also enhance the design of diagnostic tools. Additionally, an immunofluorescence test utilising patient-derived peripheral WBC serves as another valuable diagnostic method for OROV. Metagenomics is a relatively underutilised technique for detecting OROV, typically employed when conventional RT-qPCR methods fail to identify the viral OROV genome. By using metagenomic analysis of a sample from the patient, several strains of OROV have been found and identified [47]. At present, there is no recognised diagnostic standard for OROV, regardless of the detection method used discussed in (Table 2). Standardised procedures would thus be extremely beneficial in order to support the creation and validation of more trustworthy and easily available diagnostic instruments.

The time of specimen collection in relation to symptom onset has a significant impact on the accuracy of diagnosing Oropouche virus (OROV) infection. Since viral RNA levels are highest and assay specificity is at its best during the acute viremic period (usually days 1–5 of illness), molecular tests like RT-PCR or RT-qPCR are the recommended diagnostic techniques. After this window, molecular sensitivity is limited by falling viremia, and serological testing becomes more informative. However, cross-reactive antibody responses in arbovirus-endemic areas and the delayed development of IgM antibodies (usually ≥ 4–7 days post-start) complicate the interpretation of serological results [29, 43].

Tests for these diseases, such as ELISA tests that search for IgM and IgG, might produce inaccurate or ambiguous results in areas where dengue, Zika, chikungunya, and other arboviruses are prevalent at the same time. This is due to the fact that testing may mistake different arboviruses [48].

The more precise tests, such as the plaque reduction neutralization tests, are better at identifying the actual disease. These tests require two samples from the same subject, specialized tools to ensure everyone’s safety, and highly skilled personnel. These difficulties highlight how crucial it is to combine clinical context, exposure history, and test timing when choosing diagnostic strategies and analyzing test data for possible OROV infection [27].

**Table 2:** Diagnostic methods for Oropouche virus infection: principles, timing of use, performance characteristics, and key limitations

Diagnostic Method	Sample Type	Time of Use Post-Onset	Sensitivity / Specificity	Advantages	Limitations	References
RT-PCR	Blood	During peak viremia (early acute phase)	Not specified	Rapid, detects viral RNA	May not detect divergent strains; high sequence specificity required	[26]
Multiplex RT-qPCR / One-step RT-PCR	Blood	During acute phase	Not specified	Detects multiple viruses (OROV, MAYV, etc.) in one test	Not OROV-specific due to S segment conservation	[26]
Portable RT-qPCR (SmartCycle)	Blood (field use)	During outbreaks in remote areas	Not specified	Field-deployable, rapid detection in outbreaks	Requires optimization for OROV-specific detection	[27]
Serologic Testing (IgM/IgG ELISA)	Blood serum	1 day to 2 weeks post onset	Not specified	Identifies current or past infection	Cross-reactivity possible; biosafety needed for virus-derived antigens	[23, 34]
HI (Hemagglutination Inhibition)	Blood serum	Post-infection phase	Not specified	Traditional method, used in seroprevalence studies	Lower sensitivity and specificity than ELISA	[16, 24]
PRNT (Plaque Reduction Neutralization Test)	Blood serum	Post-infection phase	Not specified	Gold standard for confirming OROV antibodies	Technically demanding; requires BSL-3 facilities	[24]
Immunoassay using recombinant N protein	Blood serum	1–14 days post-onset	Sens: 95%, Spec: 99.5%	High sensitivity and specificity; avoids need for live virus	May not detect novel or reassorted strains	[25]
Immunofluorescence Assay (IFA)	Peripheral white blood cells	Variable	Not specified	Useful when viral RNA not detected by RT-PCR	Requires skilled personnel and fluorescent microscopy	[29]
Metagenomic Analysis	Blood, saliva, urine	Especially useful in unresolved cases	Not specified	Can detect divergent, reassorted, or novel strains	Costly, time-consuming, not widely available	[28]
Saliva and Urine Testing (RT-PCR)	Saliva, urine	Up to 5 days post-onset	Only 1 out of 5 tested positive	Non-invasive sampling potential	Limited evidence; not yet reliable as diagnostic tool	[22]

Table 2 lists the molecular and serological diagnostic methods for Oropouche virus (OROV) infection that are currently available. It also summarizes the best time to use each method in relation to the onset of symptoms, the reported sensitivity and specificity when available, and useful considerations for use in low-resource and outbreak situations. Each diagnostic modality’s references point to original research or reputable reviews that bolster the efficacy and use of the assay.

## Public health and prevention

### Current challenges in outbreak control

It is anticipated that the spread of OROV would have a significant effect on impacted communities. The illness was initially found in metropolitan cities and suburban areas, but it is currently extending to woodland areas. As OROV ventures into uncharted territories, it is expected that the challenges associated with healthcare systems and the economic and social health of those populations will intensify. Because they have less access to medical care and diagnostic services, rural communities may be more vulnerable, which might worsen the virus’s spread and make containment efforts more difficult [49]. Oropouche fever outbreak control now faces several obstacles, such as low diagnostic capability, underreporting since symptoms might be confused with those of other arboviruses, and low public awareness. Further complicating preventative efforts are the lack of a specialised treatment or vaccine, rising urbanisation, and growing vector populations (such *Culicoides paraensis*). Oropouche fever is difficult to diagnose because of its clinical resemblance to other feverish diseases, including dengue and Zika [50]. In order to diagnose OROV, specific laboratory testing and clinical signs are required. Although standard blood tests are not definitive, severe leukopenia-which can be as low as 2,000 leukocytes per millilitre, is frequently seen. Significant morbidity can result from recurrent OROV outbreaks, particularly in densely populated areas. These outbreaks may worsen existing public health problems and place further strain on healthcare systems and resources. Addressing the mental health effects of major outbreaks in addition to the physical health requirements of impacted persons is a problem for public health agencies. A lack of mental health supports, especially in rural or low-income areas, strains affected communities psychologically. It often causes anxiety and stress-related disorders, which strain the already overburdened healthcare system [51].

### Strategies for prevention and surveillance

The main way that the Oropouche virus infects humans is through the bite of an infected biting midge. Often referred to as “no-see-ums,” biting midges are tiny flies. It can be carried by Culex quinquefasciatus mosquitoes, which also spread St. Louis encephalitis and West Nile. There are no specific medications to treat Oropouche or immunisations to prevent it. In regions where the virus is known to exist, such as several South American, Central American, and Caribbean nations, the greatest defence against Oropouche is to avoid getting bitten by mosquitoes and midges [52].

There are several strategies to avoid OROV, including:

- Utilise insect repellents that have been certified by the Environmental Protection Agency (EPA). Insect repellents that are registered with the EPA have demonstrated safety and effectiveness when utilised according to guidelines.
- The holes in a lot of widely used window and door screens are too small to keep biting midges out. Be sure to opt for 20x20 mesh screens to keep biting midges out of your house.
- When spending time outside, utilise fans whenever you can to help drive away biting midges.
- Inform your blood center if you have received an Oropouche diagnosis recently. On the day of donation, blood donors need to be in good health.
- For at least six weeks following the onset of your OROV symptoms, you should either wear condoms or refrain from having intercourse [52].

OROV RNA has been detected in semen in limited case reports. Importantly, at least one recent study has documented replication-competent OROV isolated from semen, suggesting potential infectivity. However, documented cases of confirmed sexual transmission remain extremely limited, and epidemiologic evidence remains insufficient to quantify transmission risk. Current precautionary recommendations are therefore largely precautionary and based on analogy to other arboviruses such as Zika virus. The CDC recommendations regarding sexual precautions represent precautionary public health guidance in the context of emerging evidence rather than evidence derived from large epidemiological transmission studies. Given these findings, interim public health recommendations advise condom use or abstinence for at least six weeks following symptom resolution as a precaution, recognizing that the presence of viral RNA alone does not equate to confirmed transmissibility [53, 54].

Workers in the medical field and in laboratories who come into contact with blood, bodily fluids, or cultures from diseased people run the risk of becoming infected and should take the usual measures and follow the guidelines to reduce their exposure [55]. Although the virus has been detected in semen, its ability to transmit through intercourse is unknown. There have been no documented instances of Oropouche virus sexual transmission [56].

These precautions should be taken both while travelling and for three weeks after returning home, especially for those who are exhibiting symptoms, to stop mosquitoes or biting midges from transmitting the disease to other people in regions where they are active. Health departments are encouraged to notify the CDC via ArboNET (the national surveillance system for arthropodborne viruses) of confirmed and likely Oropouche virus infections, even though Oropouche is not a nationally notifiable condition [55]. To comprehend the danger and effectively handle the issue, it is essential to ascertain the mosquito distribution, density, and species composition over the target area. Additionally, surveillance will directly demonstrate a higher risk of Oropouche virus transmission. Underreporting and a lack of routine diagnostic tests result in limited and frequently insufficient surveillance for Oropouche fever [57]. Many cases go undiagnosed because the symptoms mimic those of other feverish diseases, such as chikungunya or dengue. Increased laboratory capacity, incorporation into national disease monitoring systems, and better case definition and reporting procedures are all necessary for effective surveillance [58]. Predicting and managing outbreaks also requires bolstering vector surveillance, particularly for *Culicoides paraensis* [6].

## Conclusion and future directions

The Oropouche virus (OROV) is an emerging global health issue that is frequently disregarded, particularly in the Caribbean and parts of South and Central America. This analysis highlights how ecological shifts, increased urbanisation, and the adaptability of the virus’s arthropod vectors-primarily *Culicoides paraensis* and some Culex species-have contributed to its expanding regional distribution. Despite improvements in diagnostic methods like RT-PCR, ELISAs, and metagenomics that have improved detection, challenges persist regarding standardisation, specificity, and access, particularly in remote and underserved areas. In the lack of targeted antiviral therapies or approved vaccines, existing prevention primarily depends on individual protective measures and vector management. Three study require immediate attention based on the experience of recent outbreaks and the needs of public health. First and foremost, in order to facilitate prompt case detection, enhance the precision of surveillance, and direct epidemic response, it is imperative that diagnostic capability be expanded and decentralized in endemic and outbreak-prone areas. Second, to stop transmission and predict geographic spread, enhanced vector surveillance and focused control measures are essential, with a focus on verified vectors like *Culicoides paraensis*. Third, to better characterize the long-term neurological aftereffects and possible prenatal consequences linked to OROV infection, longitudinal clinical and epidemiological investigations are desperately needed. Setting these areas as top priorities will improve readiness, guide evidence-based initiatives, and facilitate successful public health responses to this new arboviral threat.

Moving forward, closing these gaps necessitates a concentrated and cooperative research strategy. Main focuses involve cre Orthobunya virusating efficient vaccines and antiviral treatments, as well as designing diagnostic tools that are very sensitive, specific, and available in resource-limited environments. Thorough vector competence research is critically required to evaluate the function of both identified and potential insect vectors. Improved genomic surveillance is crucial for tracking viral evolution and identifying reassortant strains that could possess new pathogenic capabilities. Despite its significant health risks, the virology and pathogenesis of OROV remain poorly understood. Limited information on this emerging virus has largely been gleaned from the related viruses. Additionally, immediate action is required to create safe and efficient vaccinations and antivirals as well as control tactics for the midge population in metropolitan areas. More potent structure-based antiviral medications are anticipated to target important viral proteins like RdRP and the endonuclease of OBVs, including OROV, as a result of the quick development of artificial intelligence (AI) and its use in drug design. Further research is necessary to comprehend how OROV penetrates the placenta to infect neural progenitors in fetuses and how it enters the central nervous system (CNS) to cause neuronal disorders.

## Data Availability

No datasets were generated or analysed during the current study.
